# See clearer: survey on the subjective and objective information levels as well as perception and information transfer using virtual reality headsets in patients with diabetic macular edema receiving anti-VEGF treatment

**DOI:** 10.1007/s00417-022-05942-w

**Published:** 2022-12-23

**Authors:** Christian Enders, Tobias Duncker, Markus Schürks, Paula Scholz, Julia Dörner, Christian Müller, Joachim Wachtlin, Albrecht Lommatzsch

**Affiliations:** 1 MVZ Prof. Neuhann GmbH, Munich, Germany; 2 Institute of Ophthalmology, Halle, Germany; 3 Bayer Vital GmbH, Leverkusen, Germany; 4 Sankt Gertrauden Hospital, Berlin, Germany; 5 Medizinische Hochschule Brandenburg, Neuruppin, Germany; 6 Eye Center St. Franziskus Hospital, Münster, Germany

**Keywords:** Diabetic macular edema - virtual reality, Patient information, Adherence, Health literacy, Knowledge transfer

## Abstract

**Purpose:**

The purpose of this study is to investigate the use of a VR Headset in routine clinical practice as an additional source of information for patients with diabetic macular edema (DME) and their companions.

**Methods:**

Survey including 121 patients with DME, 22 companions, and 14 healthcare professionals from 8 ophthalmology centers in Germany. Patients’ and their companions’ health literacy was assessed by questionnaires including knowledge statements before and after watching a VR-based 3-D educational video. HCPs’ perspectives on the usability of a VR Headset were also assessed.

**Results:**

Patients’ mean age was 63.4 ± 12.2 years, 64.5% were men, and 76% (92/121) had previous anti-VEGF (VEGF, vascular endothelial growth factor) injections. After using the VR Headset, over 85% of patients and companions felt better informed about DME and its treatment. Patients’ mean (± SD) number of correct answers to knowledge statements increased from 13.2 ± 3.7 before to 15.5 ± 2.3 after using the VR Headset. Over 95% of patients and companions rated content and ease of understanding of the video as “very good” or “good.” Most patients and all companions considered the use of a VR Headset as a positive experience, most wishing to obtain information via VR Headset in the future. Most physicians and all medical assistants rated the effect of the VR Headset on patient satisfaction as positive and suggested further VR modules.

**Conclusion:**

After using the VR Headset, patients with DME and their companions demonstrated knowledge gains that may be meaningful individually and contribute to better adherence. This may offer an additional opportunity for knowledge transfer.

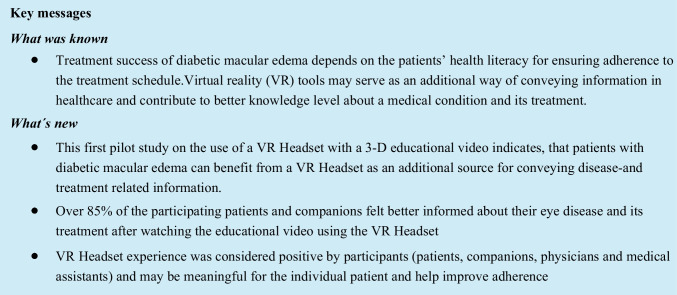

**Supplementary Information:**

The online version contains supplementary material available at 10.1007/s00417-022-05942-w.

## Introduction

Diabetic macular edema (DME), affecting approximately 5.5–7% of all patients with diabetes, is one of the most common causes of severe visual impairment and blindness in people of working age [1–3].Treatment with intravitreal injections of anti-vascular endothelial growth factors (anti-VEGF) is established as first-line therapy for DME that can maintain or even improve visual acuity [3].Successful management of this chronic disease requires consistent long-term treatment and hence high patient adherence [4], which is positively associated with patients’ health literacy [5]. However, studies found that patients with retinal diseases, including DME, exhibited low health literacy [6–8].

Offering a virtual reality (VR)-based educational video for ophthalmic patients in addition to interaction with healthcare professionals (HCPs) might improve patients’ retention and recall of information [9, 10]. A recent review, focused on cancer patients, shows that VR is suitable and acceptable as a teaching tool in healthcare and can improve patients’ health literacy [11]. In the field of ophthalmology, we are not aware of any studies on VR for patient information.

This pilot study aimed to assess patients’, companions’, and HCPs’ perceptions on the usability of the VR Headset and its impact on patients’ and companions’ knowledge level.

## Patients and methods

This survey was conducted in eight ophthalmological outpatient centers in Germany between September 2021 and February 2022. A list of participating ophthalmological centers is provided as supplementary file. Patients with DME ≥ 18 years, who were scheduled for or had already received anti-VEGF treatment and companions, were consecutively enrolled in this study. The decision to participate did not affect the patient’s medical care.

Patients and companions completed questionnaires before and after watching a specifically developed, approximately 5-min 3-D educational video on DME and its treatment via VR Headsets (Oculus Quest and Oculus Quest 2 models), which can also be used with glasses. Aspects covered were the pathophysiology of diabetic retinopathy and DME, potential symptoms of impaired vision, mode of action of intravitreal anti-VEGF agents, and importance of regular treatments. Questionnaires included questions concerning demographics, disease, treatment, and personal rating of the VR experience and specific “false/correct” knowledge statements about DME and its treatment (18 for patients and 8 for companions). After watching the video, the questionnaires had to be completed during the same office visit on the same day. Ophthalmologists and medical assistants (MA) completed separate questionnaires after watching and using the VR-video in their ophthalmological practice. The video and questionnaires were provided in German and are available as supplementary files in English.

Categorical variables were analyzed by absolute and relative frequencies, continuous variables by sample statistics (i.e., mean, standard deviation, minimum, median, quartiles, and maximum). If applicable, continuous variables were reported as an absolute value and as a change between before and after viewing the VR-video.

## Results

Questionnaires were completed by 121 patients and 22 companions before viewing the VR Headset and by all companions and all but one patient afterwards. Moreover, eight principal investigators (PI) and six MAs completed questionnaires.

### Patients

Patients’ mean (± SD) age was 63.4 ± 12.2 (range 24–87) years, with 64.5% (78/121) being men. Each of the two main age groups (< 65 years and 65 to < 80 years) included equal numbers of patients (47.1%; 57/121), while the age group of ≥ 80 years accounted for 5.8% of patients (7/121). More than half of all patients (58.7%; 71/121) were diagnosed with DME more than 12 months ago and 8.3% (10/121) within the last 3 months; for 28.9% (35/121), this information was not available. About three-quarters (76%; 92/121) of all patients had previously received anti-VEGF treatment. Of these, the majority (65%; 60/92) was diagnosed more than 12 months ago. Supplementary Table 1 provides patient characteristics.

#### Patient perceived information level

**Fig. 1:**
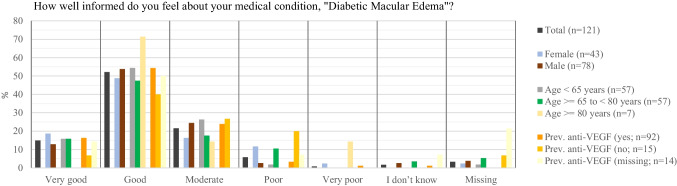
Patients’ perceived information level regarding their disease before viewing the VR-based 3-D educational video by age, sex, and previous anti-VEGF treatment

Prior to VR Headset use, patients rated their knowledge level on DME as high, with more than two-thirds ticking either “very good” (14.9%; 18/121) or “good” (52.1%; 63/121 (Fig. 1). Patients < 65 years felt slightly better informed compared to older patients aged 65 to < 80 years (“very good” and “good”: 70.2% (40/57) vs. 63.2% (36/57)). Patients previously treated with anti-VEGF felt better informed than those without treatment experience (“very good” and “good”; 70.6% (65/92) vs. 46.7% (7/15)).

**Fig. 2:**
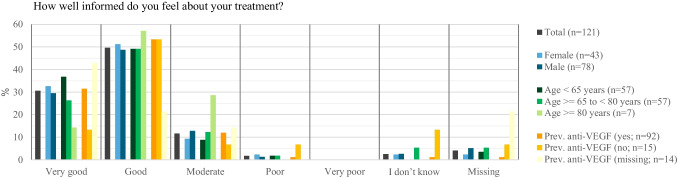
Patients’ perceived information level regarding their treatment *before* viewing the VR-based 3-D educational video by age, sex, and previous anti-VEGF treatment

A high proportion of patients (80.2%; 97/121) also considered their knowledge level regarding treatment as high, with 30.6% (37/121) rating it as “very good” and 49.6% (60/121) rating it as “good” (Fig. 2). Younger patients felt better informed, with 36.8% (21/57) of patients < 65 years choosing “very good” compared to 26.3% (15/57) in the 65 to < 80 age group. Patients with anti-VEGF treatment experience more often felt better informed than those without (“very good” and “good”; 84.8%; (88/92) vs. 66.6% (10/15)).

**Fig. 3:**
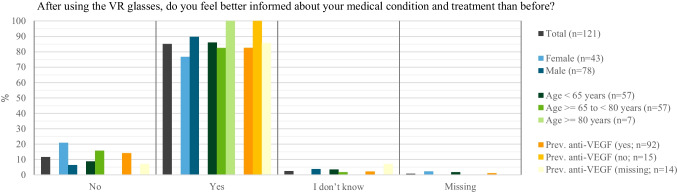
Patients’ perceived information level on DME and its treatment *after* viewing the VR-based 3-D educational video by age, sex, and previous anti-VEGF treatment

After watching the VR-video, the vast majority of patients (85.1%; 103/121) felt better informed about DME and its treatment (Fig. 3). Overall, men felt better informed than women (89.7% (70/78) vs. 76.7% (33/43)). All patients without anti-VEGF treatment experience (100%; (15/15)) felt better informed after using the VR Headset. On a scale from “0” (poorly informed) to “10” (very well informed), the mean information level score (± SD) increased for all patients with a response (*n* = 116) from 7.4 ± 2.2 to 8.4 ± 1.8 after using the VR Headset with a mean change of 1.1 ± 1.6. Results were similar between women and men, across age groups, and among patients with and without previous anti-VEGF treatment.

#### Need for information

The most important source of information for patients before using the VR Headset was the consultation with the doctor (95.9% (116/121), similar results by gender, age, and treatment experience). Internet and brochures or materials from physicians or clinic staff were each used by one-third of patients (33.1%; 40/121). Conversations with staff /receptionist were reported as information source of by 20.8% of patients (25/121). More women than men (41.9%; 18/43 vs. 28.2%; 22/78) and more younger patients used the Internet (50.9% (29/57) < 65 years vs. 17.5% (10/57) 65 to < 80 years vs. 14.3% (1/7) ≥ 80 years). Before using the VR Headset, 61.2% of patients (74/121) were not lacking information, while 19% (23/121) specified missing information. Most patients mentioned treatment options (11.6%; 14/121), followed by treatment goals (8.3%; 10/121), and consequences of the medical condition (8.3%; 10/121). After using the VR headset, 71.1% of patients (86/121) wanted to get more information about DME via VR Headset in the future, while 13.2% (16/121) did not, and 15.7% (19/121) were inconclusive or did not indicate. The most common request was for ways of influencing their own medical situation (42.1%; 51/121), followed by information on consequences of the medical condition (38.8%; 47/121) and treatment options (38.0%; 46/121).

#### Knowledge statements

**Fig. 4:**
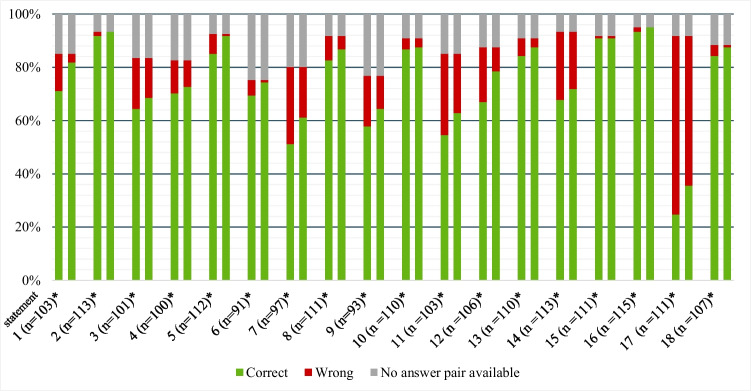
Answers (true/false) of patients to 18 knowledge statements: results before (left side of bar) and after (right side of bar) use of the VR Headset. *Each bar represents the total (*N* = 121, i.e., 100%) of patients. The respective total of patients with answer pairs (before/after) is displayed in parenthesis

For the 18 “true or false” statements (Supplementary Table 2), the number of correct responses improved from a mean (± SD) of 13.2 ± 3.7 before to 15.5 ± 2.3 after VR Headset use (Fig. 4). Small differences in the number of correct answers (before/after < 5%) were found for statements mainly concerning disease monitoring, treatment options, and specifics of the anti-VEGF treatment (statements 4, 10, 13–16, and 18, Supplementary Table 2). Larger differences in the number of correct answers (before/after > 10%) were found for statements concerning anatomy and specifics of the therapy including goals and required duration of the anti-VEGF treatment (statements 1, 7, 12, and 17; Supplementary Table 2). Results in subgroups were similar.

#### Patients’ personal rating of the VR experience

The vast majority of patients (92.6%; 112/121) considered the use of VR Headset as a positive experience. More than half of patients (55.4%; 67/121) rated content and comprehensibility of the information in the VR-based educational video as “very good,” and 43% of patients (52/121) rated it as “good.” Younger patients rather selected “very good” compared to older patients (66.7% (38/57) in < 65 years vs. 47.4% (27/57) in 65 to < 80 years vs. 28.6% (2/7) in ≥ 80 years old patients). Women considered content and comprehensibility better than men, 65.1% (28/43) of women selected “very good” vs. 50% (39/78) of men. Patients without previous anti-VEGF treatment more often reported a “very good” impression compared to those with treatment experience (60% (9/15) vs. 53.3% (49/92)). Overall, 78.5% of patients (95/121) would like to obtain additional information via VR Headset.

### Companions

Most companions (81.8%; 18/22) reported to be family members and 13.6% (3/22) stated to be friends or acquaintances. About one-third of companions each rated their patients’ level of information as “very good” (31.8%; 7/22) or “good” (36.4%; 8/22), 13.6% (3/22) rated it as “moderate,” and one companion rated it as “poor.”

#### Companions’ perceived information level

More than one-third of companions (36.4%; 8/22) rated their own information level about the patient’s medical condition and its treatment as “good,” 18.2% (4/22) as “very good,” 22.7% (5/22) as “moderate,” 9.1% (2/22) as “poor,” and 4.5% (1/22) as “very poor.” More than half of companions (54.5%; 12/22) had received their information by consultation with a doctor, 36.4% (8/22) via brochures and information material from the doctor or medical staff, 22.7% (5/22) via Internet, and 13.6% (3/22) by conversation with others. Less than half of companions (45.5%; 10/22) did not miss any information, while 36.4% (8/22) were not sure. A lack of information was perceived by 18.2% of companions (4/22). Most reported lack of information on treatment options (22.7%; 5 /22), on consequences of the disease (18.2%; 4/22), and on treatment goals (13.6%; 3/22). After VR Headset use, most companions (86.4%; 19/22) felt better informed and companions’ information level increased by in mean (± SD) 1.5 ± 3.0, from 6.7 ± 2.9 before to 8.1 ± 2.4 after using the VR Headset (scale from “0” (poorly informed) to “10” (very well informed)). Most companions (81.8%; 18/22) would like to receive more information via VR Headset regarding the patients’ medical condition (2 did not want this, 2 were unsure). Most wished information on ways to influence the disease themselves (63.6%; 14/22), treatment options (59.1%; 13/22), consequences of the medical condition (54.5%; 12/22), and how the medical condition started (45.5%; 10/22).

#### Knowledge questions

**Fig. 5:**
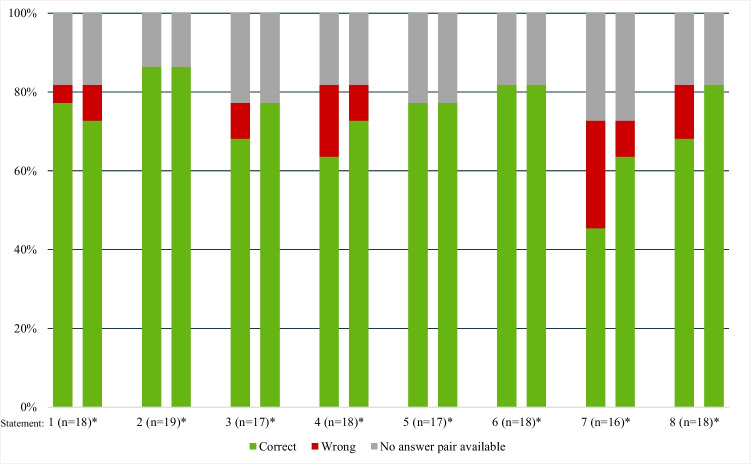
Answers (true/false) of companions to 8 knowledge statements: results before (left side of bar) and after (right side of bar) use of the VR Headset. *Each bar represents the total (*N* = 22, i.e., 100%) of companions. The respective total of companions with answer pairs (before/after) is displayed in parenthesis

Statements concerning monitoring and specifics of the anti-VEGF treatment (questions 2, 5, and 6¸ Supplementary Table 3) were answered correctly by all companions returning the questionnaire before and after using the VR Headset (Fig. 5). The largest difference (before/after) was recorded for questions about required duration of anti-VEGF treatment (25% for question 7, 11% for question 4).

#### Companions’ personal rating of the VR experience

All companions (100%; 22/22) considered the VR Headset to be a positive experience. The vast majority (90.9%; 20/22) would like to obtain information via VR Headset in the future. Over half of all companions (59.1%; 13/22) had a “very good” and 36.4% (8/22) a “good” impression of their VR experience regarding content and ease of understanding.

### Physicians

Physicians (87.5%; 7/8) mainly used VR Headsets in patients with previous anti-VEGF-treatment and considered the experience as useful. Three physicians (37.5%; 3/8) suggested its use after the doctors’ consultation, while two (25%; 2/8) favored it after the preliminary examination. All physicians stated that the VR Headset helps provide patients with information about disease and treatment and suggested additional VR modules for neovascular age-related macular degeneration (nAMD), among others. All but one physician (87.5%; 7/8) rated the effect of VR Headset on patient satisfaction with care in their practice as positive and would like to continue using VR Headsets.

### Medical assistants

All MAs considered the VR experience useful and mainly (83.3%; 5/6) used VR Headsets in previously treated patients. Half of them (50%; 3/6) suggested using the VR Headset after the consultation with the physician, the other half after the preliminary examination. All MAs found the VR Headset helpful to provide patients information about disease and treatment, rated its effect on patient satisfaction as positive, and suggested among others further VR modules for nAMD. Five MAs (83.3%; 5/6) would like to continue using VR Headset.

## Discussion

This study was the first to investigate both patients’ and their companions’ knowledge levels on DME and investigated for the first time use of a VR Headset as an additional source of information transfer in the field of ophthalmology in patients with DME.

While average disease-specific knowledge was already high at the beginning of the study, over 85% of the participating patients and companions felt better informed about their eye disease and its treatment after using the VR Headset, indicating that even visually impaired patients with DME can benefit from using a VR Headset with a 3-D educational video. We could objectify this by showing that participants answered more knowledge statements about their disease and treatment correctly after watching the VR-video. In addition, the vast majority stated that using the VR Headset was a positive experience and that they would like to receive information via this technology in the future.

The included patients represent a typical population of patients with DME receiving anti-VEGF treatment in routine clinical practice in Germany and were comparable in age and sex distribution to the cohorts of two recent studies of anti-VEGF treatment for DME in Germany and the UK [12, 13]. Overall, the information level regarding DME and anti-VEGF treatment was already high before the use of the VR Headset, both self-assessed by patients and confirmed by companions. Over two-thirds of patients already felt well or very well informed. Other studies report lower levels of information in patients treated with anti-VEGF injections for retinal diseases [6, 7]. However, these studies are not directly comparable to our study in terms of healthcare systems, patient populations, study objectives, and the questionnaires used. For example, in the study by Enders et al., 45% of all patients (*N* = 100; median age 73 years; 52% of these patients with nAMD) felt insufficiently informed despite detailed information in the medical consultation [6]. In a Danish study, 73% of DME patients (*n* = 23) rated their health literacy as poor when surveyed with the European Health Literacy Survey Questionnaire (HLS-EU-Q47) [7, 14, 15].However, this questionnaire does not include explicit questions about retinal disease or anti-VEGF treatment, which was a major reason for developing true/false knowledge statements specifically for DME patients and their companions in our study.

The high information level in our survey may be due to the majority of patients (65%) already being treated with anti-VEGF for more than 12 months, suggesting a good adherence and regular information on DME and its therapy. In particular, the high knowledge level, rated as “very good” by one-third of the patients, suggests high health literacy regarding this important topic, which is usually discussed in detail during consultations with the physicians, with room for questions from patients and companions. Further, the lower self-rated knowledge level among treatment-naïve patients compared to treatment-experienced patients suggests that treatment-naïve patients may benefit more from the educational video via the VR Headset. This will have to be addressed in future targeted studies.

Both for patients and companions, consultation with physician was the most important source of information. This is consistent with a study showing that consultation with physicians is an essential component of a successful physician–patient relationship and an important way to improve adherence [6]. As expected, brochures and the Internet were also cited as important sources of information, with the Internet being used mainly by younger patients. Conversations with practice staff or receptionists were reported only by one-fifth of patients offering some potential for improving information transfer via communication with patients and their families. However, it may also indicate that practice staff only has limited time, so digital aids like the VR Headset may be good additional information sources well received by the patients.

Our finding of a higher perceived information level in the majority of participants after using the VR Headsets is consistent with other studies [11, 16]. Although the mean knowledge gain (1.1 points for patients, 1.5 points for companions; scale of 0–10) appears small, it is important to consider the high knowledge level at baseline potentially leading to a ceiling effect. This is supported by the fact that companions, with a slightly lower baseline value (mean 6.7 for companions and 7.4 for patients), showed a greater improvement. Furthermore, even a small knowledge gain may considerably affect the individual patient’s attitude toward therapy and adherence. Notably, patients and companions achieved more pronounced improvements in statements regarding goals and duration of anti-VEGF treatment (patients: questions 1, 7, 12, 17; see Fig. 4; companions: questions 4, 7; see Fig. 5). In particular, the understanding that one single injection is not sufficient to treat DME and treatment cannot be discontinued as soon as vision has improved are crucial aspects for good long-term adherence, a key success factor in the treatment of chronic diseases. The VR-based educational video may offer added value, as the need for long-term, potentially lifelong treatment may be easier to communicate in a film than in a face-to-face conversation.

In general, there was a high level of satisfaction among participants with the VR Headset: 98.4% of patients and 95.5% of companions rated content and comprehensibility of the educational video as very good or good. The majority of patients and companions would like to receive more information via VR Headsets in the future on topics that may promote adherence and prevention. Potential modifications and upgrades are currently discussed.

The aim of this study was to investigate whether a 3-D video via a VR headset is accepted by patients as an *additional source of information* and what influence it has on patients’ health literacy. We deliberately chose a 3-D video presented via VR headset as the information vehicle, as this may give a more vivid and real-life impression on the pathophysiology and perceived symptoms. Further, it ensures local flexibility allowing patients to use it wherever they wish and may better reduce visual stimuli from outside the field of view. Hence, patients become more immersed in the simulation, which can improve retention and recall of information. Only few people perceived the VR Headset as too bulky and uncomfortable or perceived the film as too close.

Few physicians and medical assistants expressed a reluctant attitude toward the VR Headset. Reasons include the additional time burden on practice personnel and a limited suitability for older persons over 65 years.

Limitations include first, the pilot character of the study, aimed at hypothesis generation; hence, any conclusion must be drawn with caution. One such hypothesis, which is supported by our data, is that treatment-naïve patients will experience a greater benefit in terms of knowledge gain by watching the video via the VR Headset than treatment-experienced patients. This will have to be addressed in future studies. Second, the results cannot be extrapolated to DME patients in other countries due to differences in healthcare systems. In addition, results of this survey may not be representative for patients with other indications receiving anti-VEGF treatment. However, anti-VEGF treatment is the first choice in the treatment of DME, and thus the population included represents one of the most important patient groups. Third, while the VR Headset was considered a positive experience by the majority of participants, we cannot exclude a selection bias. For example, patients who considered the headset too bulky and were less tech-savvy might have been unwilling to participate.

In conclusion, VR Headset use was positively evaluated by all participants and generated knowledge gains that may be individually meaningful and may help improve adherence. Information transfer via 3-D technology with a VR Headset is feasible even for visually impaired patients with DME and may facilitate the perception of content in patients with central vision impairment. Further studies focused on treatment-naïve patients are needed to confirm these findings and to investigate if this may also be a viable approach for patients with other types of vision impairment.

## Supplementary Information

Below is the link to the electronic supplementary material.

Supplementary file1 (PDF 135 KB)

Supplementary file2 (PDF 397 KB)

Supplementary file3 (PDF 355 KB)

Supplementary file4 (PDF 273 KB)

Supplementary file5 (PDF 270 KB)

Supplementary file6 (PDF 148 KB)

Supplementary file7 (PDF 123 KB)

Supplementary file8 (PDF 121 KB)

Supplementary file9 (MP4 212581 KB)
