# Supplementary material for: See clearer: survey on the subjective and objective information levels as well as perception and information transfer using virtual reality headsets in patients with diabetic macular edema receiving anti-VEGF treatment
Source: Graefes Arch Clin Exp Ophthalmol. 2022 Dec 23;261(6):1563–70. doi: 10.1007/s00417-022-05942-w (PMC10198935; doi:10.1007/s00417-022-05942-w)
Supplement: Supplementary file 1 — Supplementary file1 (PDF 135 KB) [file 417_2022_5942_MOESM1_ESM.pdf]

Title:

See Clearer - Survey on the subjective and objective information levels as well as perception and information transfer using virtual reality headsets in patients with diabetic macular edema undergoing anti-VEGF treatment

Journal:

Graefe's Archive for Clinical and Experimental Ophthalmology

Authors:

Christian Enders, Tobias Duncker, Markus Schürks, Paula Scholz, Julia Dörner, Christian Müller, Joachim Wachtlin, Albrecht Lommatzsch

* Corresponding author

Markus Schürks

Bayer Vital GmbH, Leverkusen, Germany;

E-Mail: Markus.Schuerks@bayer.com

Orcid ID: 0000-0002-0477-8288

List of Participating ophthalmological centers:

1. Eye Center St. Franziskus Hospital, Münster, Germany
2. MVZ Prof. Neuhann GmbH, Munich, Germany
3. Institute of Ophthalmology, Halle, MVZ Ophthalmology Mitteldeutschland GmbH, Germany
4. Ophthalomogical practice Dr. Kerscher, Dr. Mihaescú, Würzburg, Germany
5. Eye Center Erkelenz, Erkelenz, Germany
6. Eye Center Frankfurt Prof. Dr. Koch GmbH, Frankfurt/Main, Germany
7. Eye practice for young and old, Berlin, Germany
8. Sankt Gertrauden Hospital GmbH, Department Ophthalmology, Berlin, Germany
