# Supplementary material for: See clearer: survey on the subjective and objective information levels as well as perception and information transfer using virtual reality headsets in patients with diabetic macular edema receiving anti-VEGF treatment
Source: Graefes Arch Clin Exp Ophthalmol. 2022 Dec 23;261(6):1563–70. doi: 10.1007/s00417-022-05942-w (PMC10198935; doi:10.1007/s00417-022-05942-w)
Supplement: Supplementary file 2 — Supplementary file2 (PDF 397 KB) [file 417_2022_5942_MOESM2_ESM.pdf]

Title:

See Clearer - Survey on the subjective and objective information levels as well as perception and information transfer using virtual reality headsets in patients with diabetic macular edema undergoing anti-VEGF treatment

Journal:

Graefe's Archive for Clinical and Experimental Ophthalmology

Authors:

Christian Enders, Tobias Duncker, Markus Schürks, Paula Scholz, Julia Dörner, Christian Müller, Joachim Wachtlin, Albrecht Lommatzsch

* Corresponding author

Markus Schürks

Bayer Vital GmbH, Leverkusen, Germany;

E-Mail: Markus.Schuerks@bayer.com

Orcid ID: 0000-0002-0477-8288

Questionnaire for patients with diabetic macular edema undergoing anti-VEGF treatment

Dear Patient,

We would like to invite you to participate in this survey of patients with diabetic macular edema. This will help us understand your level of knowledge about your illness and its treatment. We also want to investigate whether the "VR glasses" are a good way of conveying information to patients regarding their illnesses. The VR glasses ("Virtual Reality" glasses) are a helmet-like set of glasses that play a short film with a three-dimensional effect.

The goal of this test is to find new ways of conveying information to patients in an understandable and memorable way.

The survey consists of three parts in all, each of which takes about 10 minutes. To start, you will receive a questionnaire about your level of knowledge about your illness. Then there will be a short film which you will view with the VR glasses. We will then ask you to fill out a second questionnaire that is very similar to the first one. At the end of the survey, you will receive a sheet with the answers to the knowledge questions from the two questionnaires. You will also receive a 20.00€ gift certificate as our way of saying thanks for your participation.

Thank you for participating!

Date	☐ / ☐ / ☐ Day / Month / Year
Age	☐ years
Sex	☐ female ☐ male ☐ other
When was the first time you were diagnosed with diabetic macular edema?	☐ / ☐ Month / Year

Have you already received an anti-VEGF treatment injection? ☐ yes ☐ no

With the following questions, we wish to better understand how well informed you feel about your illness and its treatment.

1. How well informed do you feel **about your illness, "diabetic macular edema"**?

☐ very good ☐ good ☐ moderate ☐ poor ☐ very poor
☐ I don't know

2. How well informed do you feel **about your treatment**?

☐ very good ☐ good ☐ moderate ☐ poor ☐ very poor
☐ I don't know

3. Do you have the feeling that you **lack information on your disease or its treatment**?

☐ yes ☐ no ☐ I don't know

→ **If yes, how so?**

☐ on how the illness started
☐ on treatment options
☐ on consequences of the illness
☐ on treatment goals
☐ on ways to influence my illness myself
☐ on treatment duration
☐ on treatment frequency

☐ on the following topics → Please specify which topics:

4. Which **information sources** have you used so far regarding your illness?

- ☐ Consultation with doctor
- ☐ Conversation with medical staff / receptionist
- ☐ Brochures / informational material from doctor or medical staff
- ☐ Conversations with others
- ☐ Self-help groups
- ☐ Internet
- ☐ Magazines / newspapers
- ☐ Videos or films
- ☐ Other information sources→ Please specify which:

5. On a scale of 0 to 10, **how well informed do you feel about your illness and its treatment** (anti-VEGF therapy)**?**

Please choose the appropriate number on the scale. Here, "0" means very poorly informed and "10" means very well informed.

0	1	2	3	4	5	6	7	8	9	10

With the following questions, we'd like to better understand how much you already know about your illness and its treatment.

Below you will find 18 sentences that are either true or false.
After each sentence, please check whether you think it is true or false.

1. The macula is the point on the retina where our vision is most focused.	☐ true	☐ false
2. Diabetic retinopathy is a secondary complication of diabetes.	☐ true	☐ false
3. Diabetic macular edemas are a disease of the eye lens.	☐ true	☐ false
4. Diabetic retinopathy is a disease of the retina.	☐ true	☐ false
5. Patients with diabetic macular edema may have problems reading and recognizing faces.	☐ true	☐ false
6. The VEGF growth factor in the eye plays a major role in diabetic retinopathy and diabetic macular edema.	☐ true	☐ false
7. If the anti-VEGF treatment achieves an improvement in vision, the treatment can be discontinued.	☐ true	☐ false
8. You can treat diabetic macular edema with eye drops.	☐ true	☐ false
9. Diabetic retinopathy is a possible complication of anti-VEGF treatment.	☐ true	☐ false

10. To check treatment progress, the retina is regularly scanned with OCT (optical coherence tomography).	☐ true	☐ false
11. VEGF is a method of testing the eye.	☐ true	☐ false
12. Anti-VEGF treatment involves treating the back of the eye with a laser.	☐ true	☐ false
13. Diabetic macular edema is treated by regular dosage of a pill.	☐ true	☐ false
14. Diabetic macular edema is typically treated with a one-time injection of an anti-VEGF drug to the eye.	☐ true	☐ false
15. Anti-VEGF treatment can cause your vision to stop getting worse, or even get better.	☐ true	☐ false
16. With anti-VEGF treatment, it is important to stick to the treatment plan.	☐ true	☐ false
17. The goal of anti-VEGF treatment is to heal the diabetic macular edema.	☐ true	☐ false
18. Studies have shown that an anti-VEGF treatment can restore your ability to do certain daily activities like reading a newspaper or driving a car.	☐ true	☐ false

Questionnaire for patients with diabetic macular edema undergoing anti-VEGF treatment

The following three questions refer to the VR glasses in general.

1. Did you feel **positive about using the VR glasses**?

☐ no ☐ yes ☐ I don't know

→ If no, why not?

2. What is your **impression of the VR glasses regarding the content and ease of understanding** the information?

☐ very good ☐ good ☐ moderate☐ poor ☐ very poor I ☐ don't know

3. Do you have any **remarks about using the VR glasses** or about the technology?

With the following questions, we'd like to understand whether you feel better informed after using the VR glasses.

1. Would you like to have **knowledge from the VR glasses as an additional source of information**?

☐ no ☐ yes ☐ I don't know

→ If no, why not?

---

2. After using the VR glasses, do you feel better **informed about your illness and treatment** than before?

☐ no ☐ yes ☐ I don't know

---

3. Would you like to get **more information from VR glasses** regarding your illness in the future?

☐ no ☐ yes ☐ I don't know

→ **If yes, how so:**

☐ on how the illness started
☐ on treatment options for
☐ on consequences of the illness
☐ on treatment goals
☐ on ways to influence my illness myself
☐ on treatment duration
☐ on treatment frequency
☐ on the following topics → Please specify which topics:

---

4. On a scale of 0 to 10, how well informed do you feel about your illness and its treatment (anti-VEGF therapy)?

Please choose the appropriate number on the scale. Here, "0" means very poorly informed and "10" means very well informed.

0	1	2	3	4	5	6	7	8	9	10

With the following questions, we'd like to understand whether using the VR glasses has an effect on your level of knowledge about your illness.

Below you will find another 18 sentences that are either true or false. After each sentence, please check whether you think it is true or false.

1. The macula is the point on the retina where our vision is most focused.	☐ true	☐ false
2. Diabetic retinopathy is a secondary complication of diabetes.	☐ true	☐ false
3. Diabetic macular edemas are a disease of the eye lens.	☐ true	☐ false
4. Diabetic retinopathy is a disease of the retina.	☐ true	☐ false
5. Patients with diabetic macular edema may have problems reading and recognizing faces.	☐ true	☐ false
6. The VEGF growth factor in the eye plays a major role in diabetic retinopathy and diabetic macular edema.	☐ true	☐ false

7. If the anti-VEGF treatment achieves an improvement in vision, the treatment can be discontinued. ☐ true ☐ false

8. You can treat diabetic macular edema with eye drops. ☐ true ☐ false

9. Diabetic retinopathy is a possible complication of anti-VEGF treatment. ☐ true ☐ false

10. To check treatment progress, the retina is regularly scanned with OCT (optical coherence tomography). ☐ true ☐ false

11. VEGF is a method of testing the eye. ☐ true ☐ false

12. Anti-VEGF treatment involves treating the back of the eye with a laser. ☐ true ☐ false

13. Diabetic macular edema is treated by regular dosage of a pill. ☐ true ☐ false

14. Diabetic macular edema is typically treated with a one-time injection of an anti-VEGF drug to the eye. ☐ true ☐ false

15. Anti-VEGF treatment can cause your vision to stop getting worse, or even get better. ☐ true ☐ false

16. With anti-VEGF treatment, it is important to stick to the treatment plan. ☐ true ☐ false

17. The goal of anti-VEGF treatment is to heal the diabetic macular edema. ☐ true ☐ false

18. Studies have shown that an anti-VEGF treatment can restore your ability to do certain daily activities like reading a newspaper or driving a car. ☐ true ☐ false
