# Supplementary material for: See clearer: survey on the subjective and objective information levels as well as perception and information transfer using virtual reality headsets in patients with diabetic macular edema receiving anti-VEGF treatment
Source: Graefes Arch Clin Exp Ophthalmol. 2022 Dec 23;261(6):1563–70. doi: 10.1007/s00417-022-05942-w (PMC10198935; doi:10.1007/s00417-022-05942-w)
Supplement: Supplementary file 3 — Supplementary file3 (PDF 355 KB) [file 417_2022_5942_MOESM3_ESM.pdf]

Title:

See Clearer - Survey on the subjective and objective information levels as well as perception and information transfer using virtual reality headsets in patients with diabetic macular edema undergoing anti-VEGF treatment

Journal:

Graefe's Archive for Clinical and Experimental Ophthalmology

Authors:

Christian Enders, Tobias Duncker, Markus Schürks, Paula Scholz, Julia Dörner, Christian Müller, Joachim Wachtlin, Albrecht Lommatzsch

* Corresponding author

Markus Schürks

Bayer Vital GmbH, Leverkusen, Germany;

E-Mail: Markus.Schuerks@bayer.com

Orcid ID: 0000-0002-0477-8288

Questionnaire for companions of patients with diabetic macular edema undergoing anti-VEGF treatment

Dear companion,

as part of the survey on patients with diabetic macular edema, we are inviting you to participate as a companion of the patient. This will help us understand your level of knowledge about the patient's illness and the patient's treatment. We also want to test whether the "VR glasses" are a good way of conveying information about the illness. The VR glasses ("Virtual Reality" glasses) are a helmet-like set of glasses that play a short film with a three-dimensional effect.

The goal of this test is to find new ways of conveying information to patients in an understandable and memorable way.

The survey consists of three parts in all, each of which takes about 10 minutes. To start, you will receive a questionnaire about your level of knowledge about the patient's illness. Then there will be a short film which you will view with the VR glasses. We will then ask you to fill out a second questionnaire that is very similar to the first one. At the end of the survey, you will receive a sheet with the answers to the knowledge questions from the questionnaires for your own information. As a thank-you for your participation, you will receive a gift certificate for 20.00€.

Thank you for participating!

---

1. What is your relationship to the patient?

- ☐ Family member of the patient
- ☐ Friend/acquaintance of the patient
- ☐ Social services
- ☐ Other:

RESTRICTED

2. At what occasions do you accompany the patient?

- ☐ Eye injection treatments
- ☐ Other doctor's or ophthalmologist's appointments
- ☐ Other:
- ☐ Shopping trips
- ☐ Dealing with local authorities

3. How often do you accompany the patient **to his/her doctor's appointments**?

☐ Always ☐ usually ☐ occasionally ☐ rarely

4. **In your opinion,** how well informed is the patient about his/her illness and the treatment?

☐ very good ☐ good ☐ moderate ☐ poor ☐ very poor ☐ I don't know

With the following questions, we'd like to better understand how well informed you feel about the illness of the patient whom you are accompanying.

1. How well informed do you feel about the patient's illness and treatment of the illness?

☐ very good ☐ good ☐ moderate ☐ poor ☐ very poor ☐ I don't know

2. Do you have the feeling that you lack information about the patient's illness or about the treatment of the illness?

☐ yes ☐ no ☐ I don't know

→ If yes, how so:

- ☐ on how the illness started
- ☐ on treatment options
- ☐ on consequences of the illness
- ☐ on treatment goals
- ☐ on ways to influence the illness yourself
- ☐ on treatment duration
- ☐ on treatment frequency
- ☐ on the following topics → Please specify which topics:

RESTRICTED

3. Which information sources have you used so far regarding the patient's illness?

- ☐ Consultation with doctor
- ☐ Conversation with medical staff / receptionist
- ☐ Brochures / informational material from doctor or medical staff
- ☐ Conversations with others
- ☐ Other information sources → Please specify which:
- ☐ Self-help groups
- ☐ Internet
- ☐ Magazines / newspapers
- ☐ Videos or films

4. **On a scale of 0 to 10, how well informed do you feel about the patient's illness and planned treatment (anti-VEGF therapy)?**

   Please choose the appropriate number on the scale. Here, "0" means very poorly informed and "10" means very well informed.

0	1	2	3	4	5	6	7	8	9	10

RESTRICTED

With the following questions, we'd like to better understand how well informed you feel about the patient's treatment.

Below you will find 8 sentences that are either true or false.

After each sentence, please check whether you think it is true or false.

1.	You can treat diabetic macular edema with eye drops and pills.	☐ true	☐ false
2.	To check treatment progress, the retina is regularly scanned with OCT (optical coherence tomography).	☐ true	☐ false
3.	Anti-VEGF treatment involves treating the back of the eye with a laser.	☐ true	☐ false
4.	Diabetic macular edema is typically treated with a one-time injection of an anti-VEGF drug to the eye.	☐ true	☐ false
5.	Anti-VEGF treatment can cause your vision to stop getting worse, or even get better.	☐ true	☐ false
6.	With anti-VEGF treatment, it is important to stick to the treatment plan.	☐ true	☐ false
7.	If the anti-VEGF treatment achieves an improvement in vision, the treatment can be discontinued.	☐ true	☐ false
8.	Studies have shown that an anti-VEGF treatment can restore your ability to do certain daily activities like reading a newspaper or driving a car.	☐ true	☐ false

RESTRICTED

Questionnaire for companions of patients with diabetic macular edema undergoing anti-VEGF treatment

1. Did you feel positive about **using the VR glasses**?

   ☐ no ☐ yes ☐ I don't know

   → If no, why not?

2. What is your **impression of the VR glasses** regarding the content and ease of understanding the information?

   ☐ very good ☐ good ☐ moderate ☐ poor ☐ very poor ☐ I don't know

3. Do you have any remarks about using the VR glasses or about the technology?

With the following questions, we'd like to understand how you view the use of the VR glasses in comparison to other information sources.

The following refer to you as the patient's companion

1. Would you like to have knowledge from the VR glasses as an additional source of information?

☐ no ☐ yes ☐ I don't know

→ If no, why not?

---

2. After using the VR glasses, do you feel better informed about the patient's illness and treatment than you did before?

☐ no ☐ yes ☐ I don't know

---

3. Would you like to get more information from VR glasses regarding the patient's illness in the future?

☐ no ☐ yes ☐ I don't know

→ If yes, how so?

☐ on how the illness started
☐ on treatment options
☐ on consequences of the illness
☐ on treatment goals
☐ on ways to influence the illness yourself
☐ on treatment duration
☐ on treatment frequency
☐ on the following topics → Please specify which topics:

---

4. **On a scale of 0 to 10, how well informed do you feel about the patient's illness and planned treatment (anti-VEGF therapy)?**

   Please choose the appropriate number on the scale. Here, "0" means very poorly informed and "10" means very well informed.

0	1	2	3	4	5	6	7	8	9	10

With the following questions, we'd like to understand whether using the VR glasses has an influence on how well informed you are about the patient's treatment.

Below you will find 8 sentences that are either true or false.

After each sentence, please check whether you think it is true or false.

1. You can treat diabetic macular edema with eye drops and pills.	☐ true	☐ false
2. To check treatment progress, the retina is regularly scanned with OCT (optical coherence tomography).	☐ true	☐ false
3. Anti-VEGF treatment involves treating the back of the eye with a laser.	☐ true	☐ false
4. Diabetic macular edema is typically treated with a one-time injection of an anti-VEGF drug to the eye.	☐ true	☐ false
5. Anti-VEGF treatment can cause your vision to stop getting worse, or even get better.	☐ true	☐ false
6. With anti-VEGF treatment, it is important to stick to the treatment plan.	☐ true	☐ false
7. If the anti-VEGF treatment achieves an improvement in vision, the treatment can be discontinued.	☐ true	☐ false
8. Studies have shown that an anti-VEGF treatment can restore your ability to do certain daily activities like reading a newspaper or driving a car.	☐ true	☐ false
