# Supplementary material for: See clearer: survey on the subjective and objective information levels as well as perception and information transfer using virtual reality headsets in patients with diabetic macular edema receiving anti-VEGF treatment
Source: Graefes Arch Clin Exp Ophthalmol. 2022 Dec 23;261(6):1563–70. doi: 10.1007/s00417-022-05942-w (PMC10198935; doi:10.1007/s00417-022-05942-w)
Supplement: Supplementary file 4 — Supplementary file4 (PDF 273 KB) [file 417_2022_5942_MOESM4_ESM.pdf]

Title:

See Clearer - Survey on the subjective and objective information levels as well as perception and information transfer using virtual reality headsets in patients with diabetic macular edema undergoing anti-VEGF treatment

Journal:

Graefe's Archive for Clinical and Experimental Ophthalmology

Authors:

Christian Enders, Tobias Duncker, Markus Schürks, Paula Scholz, Julia Dörner, Christian Müller, Joachim Wachtlin, Albrecht Lommatzsch

* Corresponding author

Markus Schürks

Bayer Vital GmbH, Leverkusen, Germany;

E-Mail: Markus.Schuerks@bayer.com

Orcid ID: 0000-0002-0477-8288

Questionnaire for ophthalmologists on the use of VR glasses in their practice

To be filled out at the end of the patient survey and after the physician being surveyed has tested the VR glasses himself/herself at least once.

With the following questions, we'd like to better understand your view of the VR glasses and their use in your practice.

1. **With how many patients** have you used the VR glasses? (approx. %)

   a. Treatment-naive DME patients ☐ %

   b. Previously treated DME patients, up to 6 months in treatment ☐ %

   c. Previously treated DME patients, >6 months in treatment ☐ %

2. **At what point in time** should the glasses be used, in your opinion?

   - ☐ After registering at reception
   - ☐ After preliminary exam by medical assistant
   - ☐ After doctor consultation
   - ☐ Other point in time:

3. **Where** were the glasses typically used? (multiple selection possible)

   - ☐ In the waiting room
   - ☐ In the doctor's office
   - ☐ In the preliminary exam room
   - ☐ In a spare room (e.g. OCT or laser room)
   - ☐ Other area:

4. **Who** assisted the patient in using the VR glasses?

   - ☐ Medical assistant
   - ☐ Physician

RESTRICTED

5. Did you find the glasses **useful**?

☐ no ☐ yes ☐ I can't say yet

→ If no, why not?

6. Can the VR glasses help you in **conveying information** to your patients regarding their illness and treatment?

☐ no ☐ yes ☐ I can't say yet

→ If no, why not?

7. What do you think of the effect of the VR glasses on the **patients' satisfaction** with the care they receive in your practice?

☐ positive ☐ no effect ☐ negative ☐ I can't say yet

8. Do you want to **continue using** the VR glasses?

☐ no ☐ yes ☐ I can't say yet

→ If no, why not?

RESTRICTED

9. Is there anything that could be **changed/improved** that would make you find the glasses useful (or more useful)?

   - ☐ Additional modules for DME/DR
   - ☐ Additional modules for nAMD
   - ☐ Additional languages:
     - ☐ English
     - ☐ Polish
     - ☐ Arabic
     - ☐ Turkish
     - ☐ Russian
     - ☐ Other languages:
   - ☐ Other:

10. Do you have any remarks about using the VR glasses or about the technology?

11. Would you be willing to **purchase** a set of VR glasses for your practice/clinic for the purpose of patient information (the price is currently approx. 350 Euros)?

    ☐ no ☐ yes ☐ I can't say yet

    → If no, would you be willing to purchase glasses if there were data that showed that patients find this type of information useful?

    ☐ no ☐ yes ☐ I can't say yet

RESTRICTED

12. Would **one set of VR glasses** be enough, in your opinion?

☐ no ☐ yes ☐ I can't say yet

→ If not, how many VR glasses would you need?

13. Are there **other conditions** which, in your opinion, would have to be met in order to routinely use the VR glasses in your practice?

☐ no ☐ yes ☐ I can't say yet

→If yes, what other conditions would have to be met?

14. Do you have any further comments?

Location, date

Physician signature

RESTRICTED
