# Supplementary material for: See clearer: survey on the subjective and objective information levels as well as perception and information transfer using virtual reality headsets in patients with diabetic macular edema receiving anti-VEGF treatment
Source: Graefes Arch Clin Exp Ophthalmol. 2022 Dec 23;261(6):1563–70. doi: 10.1007/s00417-022-05942-w (PMC10198935; doi:10.1007/s00417-022-05942-w)
Supplement: Supplementary file 5 — Supplementary file5 (PDF 270 KB) [file 417_2022_5942_MOESM5_ESM.pdf]

Title:

See Clearer - Survey on the subjective and objective information levels as well as perception and information transfer using virtual reality headsets in patients with diabetic macular edema undergoing anti-VEGF treatment

Journal:

Graefe's Archive for Clinical and Experimental Ophthalmology

Authors:

Christian Enders, Tobias Duncker, Markus Schürks, Paula Scholz, Julia Dörner, Christian Müller, Joachim Wachtlin, Albrecht Lommatzsch

* Corresponding author

Markus Schürks

Bayer Vital GmbH, Leverkusen, Germany;

E-Mail: Markus.Schuerks@bayer.com

Orcid ID: 0000-0002-0477-8288

Questionnaire for medical assistants on the use of the VR glasses in a [illegible]

To be filled out at the end of the patient survey and after the medical assistant being surveyed has tested the VR glasses himself/herself at least once.

With the following questions, we'd like to better understand your opinion about the VR glasses and their use in a practice setting.

1. **For what patient groups** can the glasses best be used, in your opinion?

☐ **Treatment-naive** patients with diabetic macular edema (DME)
☐ Previously treated DME patients having undergone **treatment for up to 6 months**
☐ Previously treated DME patients having undergone **treatment for more than 6 months**

2. **At what point in time** should the glasses be used, in your opinion?

☐ After registering at reception
☐ After preliminary exam by medical assistant
☐ After doctor consultation
☐ Other point in time:

3. **Where** were the glasses typically used? (multiple selection possible)

☐ In the waiting room
☐ In the preliminary exam room
☐ In the doctor's office
☐ In a spare room (e.g. OCT or laser room)
☐ Other area:

4. **Who** assisted the patient in using the VR glasses?

☐ Medical assistant
☐ Physician

Do you have any comments?

5. Did you find the glasses **useful**?

☐ no ☐ yes ☐ I can't say yet

→ If no, why not?

6. Can the VR glasses help you in **conveying information** to your patients regarding their illness and treatment?

☐ no ☐ yes ☐ I can't say yet

→ If no, why not?

7. What do you think of the effect of the VR glasses on the **patients' satisfaction** with the care they receive in the practice?

☐ positive ☐ no effect ☐ negative ☐ I can't say yet

8. Do you want to **continue using** the VR glasses?

☐ no ☐ yes ☐ I can't say yet

→ If no, why not?

9. Is there anything that could be **changed/improved** that would make you find the glasses useful (or more useful)?

- ☐ Additional modules for diabetic macular edema (DME) / Diabetic retinopathy (DR)
- ☐ Additional modules for (non) age-related macular degeneration (nAMD / AMD)
- ☐ Additional languages:
  - ☐ English
  - ☐ Polish
  - ☐ Arabic
  - ☐ Turkish
  - ☐ Russian
  - ☐ Other languages:
- ☐ Other:

10. Do you have any remarks about using the VR glasses or about the technology?

11. If VR glasses were available in the practice, would one set of VR glasses be enough in your opinion?

☐ no ☐ yes ☐ I can't say yet

→ If not, how many VR glasses would you need?

12. Are there **other conditions** which, in your opinion, would have to be met in order to routinely use the VR glasses in the practice?

☐ no ☐ yes ☐ I can't say yet

→ If yes, what other conditions would have to be met?

13. Do you have any further comments?
