# Supplementary material for: See clearer: survey on the subjective and objective information levels as well as perception and information transfer using virtual reality headsets in patients with diabetic macular edema receiving anti-VEGF treatment
Source: Graefes Arch Clin Exp Ophthalmol. 2022 Dec 23;261(6):1563–70. doi: 10.1007/s00417-022-05942-w (PMC10198935; doi:10.1007/s00417-022-05942-w)
Supplement: Supplementary file 6 — Supplementary file6 (PDF 148 KB) [file 417_2022_5942_MOESM6_ESM.pdf]

Title:

See Clearer - Survey on the subjective and objective information levels as well as perception and information transfer using virtual reality headsets in patients with diabetic macular edema undergoing anti-VEGF treatment

Journal:

Graefe's Archive for Clinical and Experimental Ophthalmology

Authors:

Christian Enders, Tobias Duncker, Markus Schürks, Paula Scholz, Julia Dörner, Christian Müller, Joachim Wachtlin, Albrecht Lommatzsch

* Corresponding author

Markus Schürks

Bayer Vital GmbH, Leverkusen, Germany;

E-Mail: Markus.Schuerks@bayer.com

Orcid ID: 0000-0002-0477-8288

Supplementary Table 1: Patients´ characteristics.

Supplementary Table 1: Patients´ characteristics.

	**Total** (N=121)
**Age, years**	
Mean (±SD)	63.4 (±12.2)
Median<br>(Q25% - Q75%)	65.0<br>(58.0 – 70.0)
Min. - Max.	24 – 87
**Age Groups, n (%)**	
< 65 years	57 (47.1)
65 to < 80 years	57 (47.1)
>= 80 years	7 (5.8)
**Sex, n (%)**	
Female	43 (35.5)
Male	78 (64.5)
**Time since diagnosis, n (%)**	
< 3 months	10 (8.3)
3 – 12 months	5 (4.1)
> 12 months	71 (58.7)
Missing	35 (28.9)
**Previous anti-VEGF injection, n (%)**	
Yes	92 (76.0)
No	15 (12.4)
Missing	14 (11.6)

SD, standard deviation; VEGF, vascular endothelial growth factor
