# Supplementary material for: See clearer: survey on the subjective and objective information levels as well as perception and information transfer using virtual reality headsets in patients with diabetic macular edema receiving anti-VEGF treatment
Source: Graefes Arch Clin Exp Ophthalmol. 2022 Dec 23;261(6):1563–70. doi: 10.1007/s00417-022-05942-w (PMC10198935; doi:10.1007/s00417-022-05942-w)
Supplement: Supplementary file 7 — Supplementary file7 (PDF 123 KB) [file 417_2022_5942_MOESM7_ESM.pdf]

Title:

See Clearer - Survey on the subjective and objective information levels as well as perception and information transfer using virtual reality headsets in patients with diabetic macular edema undergoing anti-VEGF treatment

Journal:

Graefe's Archive for Clinical and Experimental Ophthalmology

Authors:

Christian Enders, Tobias Duncker, Markus Schürks, Paula Scholz, Julia Dörner, Christian Müller, Joachim Wachtlin, Albrecht Lommatzsch

* Corresponding author

Markus Schürks

Bayer Vital GmbH, Leverkusen, Germany;

E-Mail: Markus.Schuerks@bayer.com

Orcid ID: 0000-0002-0477-8288

Supplementary Table 2: Eighteen statements about medical condition and treatment which patients had to answer (true/false) before and after using the VR Headset.

Supplementary Table 2: Eighteen statements about medical condition and treatment which patients had to answer (true/false) before and after using the VR Headset.

No.	Statement
1.	The macula is the point on the retina where our vision is most focused.
2.	Diabetic retinopathy is a secondary complication of diabetes.
3.	Diabetic macular edemas are a disease of the eye lens.
4.	Diabetic retinopathy is a disease of the retina.
5.	Patients with diabetic macular edema may have problems reading and recognizing faces.
6.	The VEGF growth factor in the eye plays a major role in diabetic retinopathy and diabetic macular edema.
7.	If the anti-VEGF treatment achieves an improvement in vision, the treatment can be discontinued.
8.	You can treat diabetic macular edema with eye drops.
9.	Diabetic retinopathy is a possible complication of anti-VEGF treatment.
10.	To check treatment progress, the retina is regularly scanned with OCT (optical coherence tomography).
11.	VEGF is a method of examining the eye.
12.	Anti-VEGF treatment involves treating the back of the eye with a laser.
13.	Diabetic macular edema is treated by regular intake of a pill.
14.	Diabetic macular edema is typically treated with a one-time injection of an anti-VEGF drug into the eye.
15.	Anti-VEGF treatment can cause your vision to stop getting worse, or even get better.
16.	With anti-VEGF treatment, it is important to stick to the treatment plan.
17.	The goal of anti-VEGF treatment is to heal the diabetic macular edema.
18.	Studies have shown that an anti-VEGF treatment can restore your ability to do certain daily activities like reading a newspaper or driving a car.
