# Supplementary material for: See clearer: survey on the subjective and objective information levels as well as perception and information transfer using virtual reality headsets in patients with diabetic macular edema receiving anti-VEGF treatment
Source: Graefes Arch Clin Exp Ophthalmol. 2022 Dec 23;261(6):1563–70. doi: 10.1007/s00417-022-05942-w (PMC10198935; doi:10.1007/s00417-022-05942-w)
Supplement: Supplementary file 8 — Supplementary file8 (PDF 121 KB) [file 417_2022_5942_MOESM8_ESM.pdf]

Title:

See Clearer - Survey on the subjective and objective information levels as well as perception and information transfer using virtual reality headsets in patients with diabetic macular edema undergoing anti-VEGF treatment

Journal:

Graefe's Archive for Clinical and Experimental Ophthalmology

Authors:

Christian Enders, Tobias Duncker, Markus Schürks, Paula Scholz, Julia Dörner, Christian Müller, Joachim Wachtlin, Albrecht Lommatzsch

* Corresponding author

Markus Schürks

Bayer Vital GmbH, Leverkusen, Germany;

E-Mail: Markus.Schuerks@bayer.com

Orcid ID: 0000-0002-0477-8288

Table 3: Statements about medical condition and treatment which companions had to answer (true/false) before and after using the VR Headset.

No.	Statement
1.	You can treat diabetic macular edema with eye drops and pills.
2.	To check treatment progress, the retina is regularly scanned with OCT (optical coherence tomography).
3.	Anti-VEGF treatment involves treating the back of the eye with a laser.
4.	Diabetic macular edema is typically treated with a one-time injection of an anti-VEGF drug to the eye.
5.	Anti-VEGF treatment can cause your vision to stop getting worse, or even get better.
6.	With anti-VEGF treatment, it is important to stick to the treatment plan.
7.	If the anti-VEGF treatment achieves an improvement in vision, the treatment can be discontinued
8.	Studies have shown that an anti-VEGF treatment can restore your ability to do certain daily activities like reading a newspaper or driving a car.
